# Diversity and functions of bacterial community in drinking water biofilms revealed by high-throughput sequencing

**DOI:** 10.1038/srep10044

**Published:** 2015-06-12

**Authors:** Yuanqing Chao, Yanping Mao, Zhiping Wang, Tong Zhang

**Affiliations:** 1 Environmental Biotechnology Laboratory, The University of Hong Kong, Pokfulam Road, Hong Kong SAR, China; 2 School of Environmental Science and Engineering, Sun Yat-Sen University, Guangzhou, China; 3 School of Environmental Science and Engineering, Shanghai Jiao Tong University, Shanghai, China

## Abstract

The development of biofilms in drinking water (DW) systems may cause various problems to water quality. To investigate the community structure of biofilms on different pipe materials and the global/specific metabolic functions of DW biofilms, PCR-based 454 pyrosequencing data for 16S rRNA genes and Illumina metagenomic data were generated and analysed. Considerable differences in bacterial diversity and taxonomic structure were identified between biofilms formed on stainless steel and biofilms formed on plastics, indicating that the metallic materials facilitate the formation of higher diversity biofilms. Moreover, variations in several dominant genera were observed during biofilm formation. Based on PCA analysis, the global functions in the DW biofilms were similar to other DW metagenomes. Beyond the global functions, the occurrences and abundances of specific protective genes involved in the glutathione metabolism, the SoxRS system, the OxyR system, RpoS regulated genes, and the production/degradation of extracellular polymeric substances were also evaluated. A near-complete and low-contamination draft genome was constructed from the metagenome of the DW biofilm, based on the coverage and tetranucleotide frequencies, and identified as a *Bradyrhizobiaceae*-like bacterium according to a phylogenetic analysis. Our findings provide new insight into DW biofilms, especially in terms of their metabolic functions.

It is widely accepted that biofilms constitute a remarkable portion of the microorganisms in drinking water distribution systems (DWDS)^1^,^2^. The development of biofilms provides numerous advantages to the embedded bacteria, including increases in bacterial resistance to various environmental stresses, sharing of nutrients and metabolic products among bacteria, and facilitation of horizontal gene transfer within the biofilm community^3^,^4^. Consequently, pathogens in biofilms may potentially survive residual disinfectants and proliferate to higher abundances under oligotrophic conditions in DWDS, imposing a threat to public health^4^. Several other significant problems may also be related to the development of biofilms in DWDS, including corrosion of pipes^5^, consumption of residual disinfectants^6^, and generation of tastes and odors^7^. Thus, great concern has been focused on the prevention or removal of biofilms to improve water safety and water quality in DWDS.

Previous studies of microbial communities in drinking water (DW) biofilms have utilized various molecular techniques, such as DGGE^8^, FISH^9^, T-RFLP^10^, clone libraries^11^, and microarrays^12^. These studies have provided useful information regarding microbial community structures in DW biofilms and evaluated the influences of relevant environmental factors and water characteristics, including water temperature, pH, pipe materials, disinfectant concentration, and flow rates^13^. Recently, high-throughput sequencing (HTS) techniques have demonstrated considerable advantages for the analysis of the microbial communities in association with the unprecedented sequencing depth^14^ and have been widely applied to investigations of microbial community structure in various complex environments, including DWDS biofilms^6^,^15^. These studies have successfully assessed the microbial community structure in DW biofilms at a high resolution. Unfortunately, relevant factors, such as the biofilm age or pipe material, were not evaluated in these studies. Moreover, the bacterial functions of the community, which may be crucial for comprehensively understanding DW biofilms, were not evaluated.

The aim of the present study was to address the following questions: (1) What is the impact of the type of pipe materials on biofilm development and community structure? (2) What are the functions that are highly relevant to the DW biofilms determined by comparison with other typical aquatic ecosystems? (3) What are the key protective functions that play roles in the development of DW biofilms under environmental exposure to residual disinfectants? To answer these questions, DW biofilms were cultivated on various types of materials in annular reactors in tap water. PCR-based 454 pyrosequencing data and Illumina metagenomic data were generated and analysed (1) to investigate the taxonomic differences among DW biofilms that formed on different types of materials; (2) to evaluate taxonomic changes in microbial community structure during biofilm formation over a 180 day period; (3) to compare the global functions of DW biofilms with other typical aquatic metagenomes by using principal component analysis (PCA); and (4) to characterize key protective functions, e.g., GSH metabolism, the SoxRS system, the OxyR system, RpoS regulated genes, and EPS production/degradation, in DW biofilms.

## Results

### Taxonomic analysis

**Figure 1:**
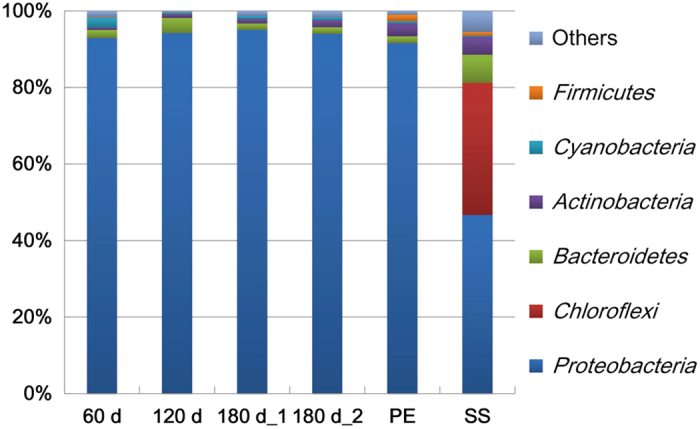
Relative distribution of 454 reads at the phylum level, as classified by RDP Classifier at a confidence threshold of 80%, for the 6 DW biofilm samples. The number of reads that were annotated to the *Bacteria* domain was designated as 100%.

**Figure 2:**
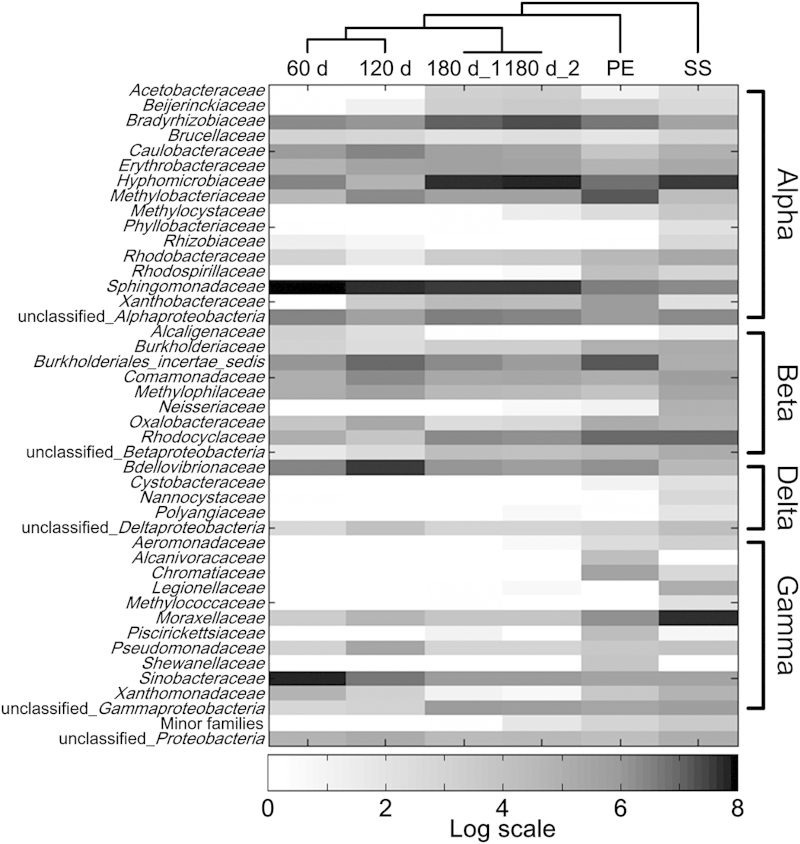
Relative distribution of 454 reads among families of the *Proteobacteria* phylum, classified by RDP Classifier, for the 6 DW biofilm samples. The families that accounted for greater than 0.5% of reads in at least one sample are shown in the figure. The clustering among samples was generated according to Gower distance using the PAST software (v 1.99).

To characterize the microbial community structure of the DW biofilms, a taxonomic analysis was conducted using the RDP Classifier to identify the 454 reads of the DW biofilms. The results indicate that *Proteobacteria* had the highest abundance on both of the plastic and metallic materials (Fig. 1), accounting for 86 ± 19% of the reads in the biofilms analysed. Compared with the plastics (92-95% *Proteobacteria*), the biofilms that formed on SS had a lower abundance of *Proteobacteria* (47%) whereas other phyla, e.g., *Chloroflexi* and *Bacteroidetes*, had a relatively higher abundance. Similar results were observed when evaluating the diversity (Table 1) and rarefaction curves (Fig. S1) of the biofilms on the two materials, suggesting that SS might facilitate the formation of a more diverse biofilm in DW systems, compared with plastics. The bacterial structure of the *Proteobacteria* phylum was further analysed at deeper levels. The *Alphaproteobacteria* was the class with the highest abundance of reads (56 ± 14%) in all biofilms (Fig. 2), followed by the *Betaproteobacteria* (19 ± 7.6%) and the *Gammaproteobacteria* (16 ± 9.5%).

**Figure 3:**
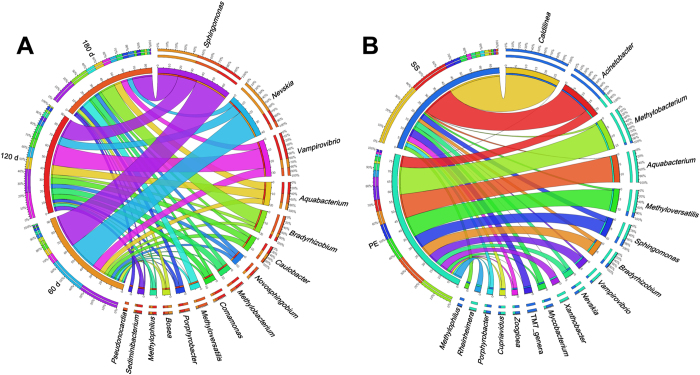
Top 10 most abundant genera in the 60, 120, and 180 d (A), and the PE and SS (B) biofilm samples. The left segments represent the DW biofilm samples, and the right segments represent the top 10 genera in each biofilm. In the inner layer, each different colour represents a different sample or genus, and the numbers beside the axis indicate the relative abundances of the corresponding samples or genera. The width of the ribbons connecting the sample and genus segments indicates the relative abundances of the corresponding genera in the accordant biofilm samples. In the outer layer of the arc for the sample segments, the different colours represent the top 10 most abundant genera in each sample, with the percentages of reads presented in descending order. For the genus segments, the outer layer illustrates the distribution of the specific genera in each DW biofilm sample.

Although the bacterial diversities of the 60, 120 and 180 d biofilms did not vary largely (Table 1), the taxonomic analysis indicated that the structure of the bacterial community changed during the biofilm formation and development. At 60 d, the genus *Sphingomonas* (in the *Sphingomonadaceae* family) and the genus *Nevskia* (in the *Sinobacteraceae* family) represented the two most abundant genera (Figs. 2 and 3). However, their relative abundance decreased considerably in the older biofilms. The decrease in *Sphingomonas* was consistent with a previous report that this genus was only dominant at the early stage of a biofilm^16^. For *Nevskia*, such variation has not been previously observed. Several bacteria, e.g., member of the family *Hyphomicrobiaceae* and the genus *Bradyrhizobium*, increased during biofilm cultivation (Figs. 2 and 3). Although the majority of these bacteria have been observed in previous studies of DW biofilms, this study revealed the dynamics of these bacteria during biofilm development, which has not been previously reported. Moreover, a significant effect of pipe material on the microbial community structure of the DW biofilms was observed in the present study (Fig. 3), implying that the type of pipe material might be a key feature governing the structure of DW biofilm communities. For instance, although *Sphingomonas*, *Nevskia* and *Vampirovibrio* were the top 3 most abundant bacteria on the plastic surface, their abundances decreased considerably during the formation and development of the biofilm community on the SS surface. Several genera, including *Vampirovibrio*, *Pseudonocardia* and *Rheinheimera* (Fig. 3), were detected in DW biofilms for the first time, demonstrating the natural complexity of DW biofilms and the limitations of our understanding of bacterial communities in DW biofilms.

Several specific bacterial groups, including potentially pathogenic bacteria, iron-oxidizing bacteria (IOB), and sulfate-reducing bacteria (SRB), were investigated (Supplemental Table S1). For potentially pathogenic bacteria, 6 genera could be detected in DW biofilms (Fig. S2). *Pseudomonas* was the most abundant genus, followed by *Mycobacterium* and *Legionella*. This finding is in agreement with other studies^6^,^8^,^17^, implying that DW biofilms harbouring opportunistic pathogens are a common issue. IOB and SRB were also investigated because they are often involved in pipe corrosion in DWDS^5^. Two bacterial genera (*Rhodobacter* and *Clostridium*) that contain IOB and SRB were detected at very low abundances in the DW biofilms (Figure S2). Considering that *Rhodobacter* are typically photosynthetic bacteria^18^ and that *Clostridium* are obligate anaerobic bacteria, the dark environment and high DO concentration (Table 2) in DWDS may inhibit their activity. Thus the pipe corrosion caused by IOB and/or SRB may not be a serious problem in our DWDS.

### Global functions of DW biofilms

**Figure 4:**
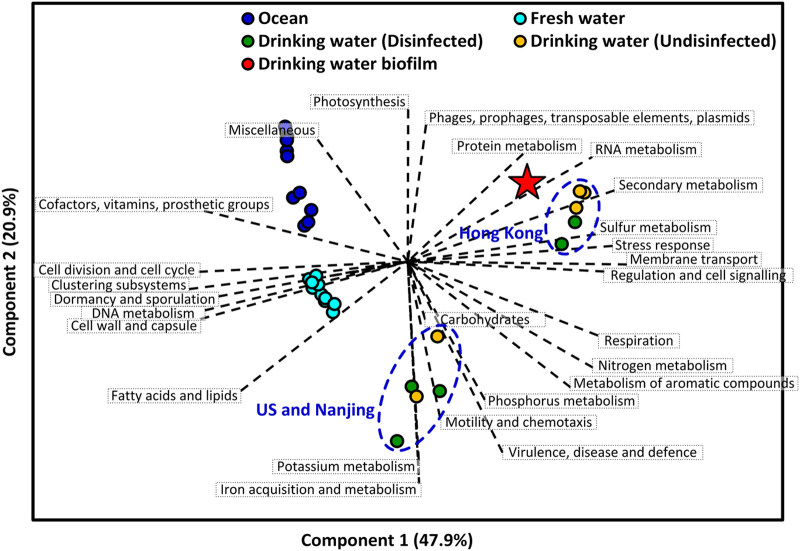
Principal component analysis of 3 typical aquatic ecosystems according to the percentage of annotated reads/tags in the SEED subsystems. The red star indicates the DW biofilm metagenome analysed in the present study. The metagenomes of the ocean, fresh water and DW samples were analysed by using the publicly available data on MG-RAST. The information for these ecosystems is provided in Table S6.

To comprehensively compare the DW biofilm to those of other typical aquatic ecosystems and to identify specific functional attributes of the DW biofilm, a PCA analysis of multiple aquatic ecosystems was conducted. In the PCA plot (Fig. 4), the metagenomes for the ecosystems are differentiated, indicating that considerable functional differences existed amongst aquatic ecosystems. However, considering the DW metagenomes, two sub-clusters could be found, one for the Hong Kong DW samples and the other for the US and Nanjing samples. One possible reason for this distribution might be the difference in climate zones among sample locations; Hong Kong is under a tropical climate, whereas the other DW samples were collected from temperate zones. Interestingly, the DW samples collected before and after disinfection did not differ significantly in the PCA plot. This result is consistent with our previous observation that the employed disinfection processes do not significantly influence the basic functions of DW microorganisms^19^. The metagenome of the DW biofilm clustered closely to the Hong Kong DW samples, revealing that the environmental factors might affect community functions to a greater extent than the mode of microbial growth (e.g., suspended or attached growth in a DW system).

### Protective functions against residual disinfectants

**Figure 5:**
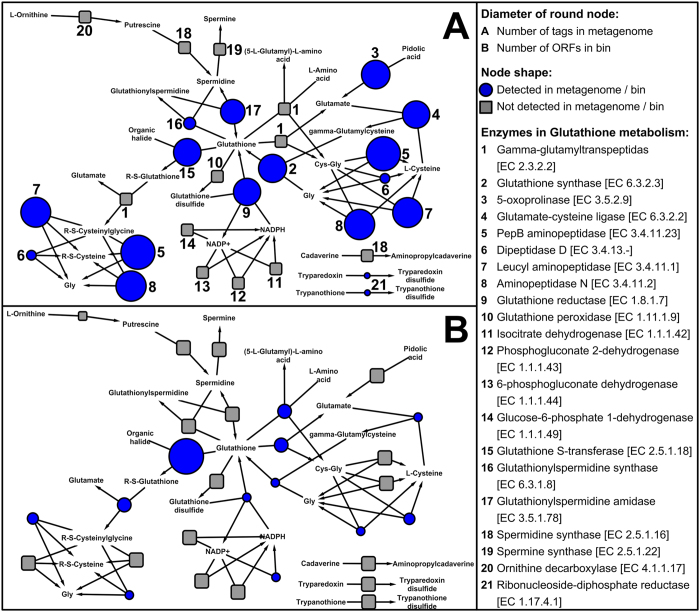
KEGG network of GSH metabolism, constructed by using 1,205 bacterial species (non-redundant) in KEGG. Network A contains the abundances of annotated enzymes detected in the DW biofilm metagenome. Network B contains the numbers of annotated enzymes (ORFs) detected in the draft genome.

The pathway for GSH metabolism was reorganized by using 1,205 (non-redundant) bacterial species contained in KEGG^20^, because GSH has been proven to play crucial roles in the survival of DW microbiomes attributed to advantages in terms of resistance against various disinfectants^19^,^21^. Compared with the 40 enzymes in the original KEGG pathway, the reconstructed pathway for bacteria contained 21 enzymes (Fig. 5). In the DW biofilm metagenome, only 12 enzymes were detected (Fig. 5A), implying that the bacteria in the DW biofilm contained a partial pathway for GSH synthesis and degradation. According to the metagenomic data, all three of the enzymes for bacterial GSH synthesis, including glutathione synthase, glutathione reductase and glutathionylspermidine amidase, were detected at considerable abundances (Fig. 5A). This result suggests that DW biofilm was capable of synthesizing GSH vigorously via multiple reactions.

**Figure 6:**
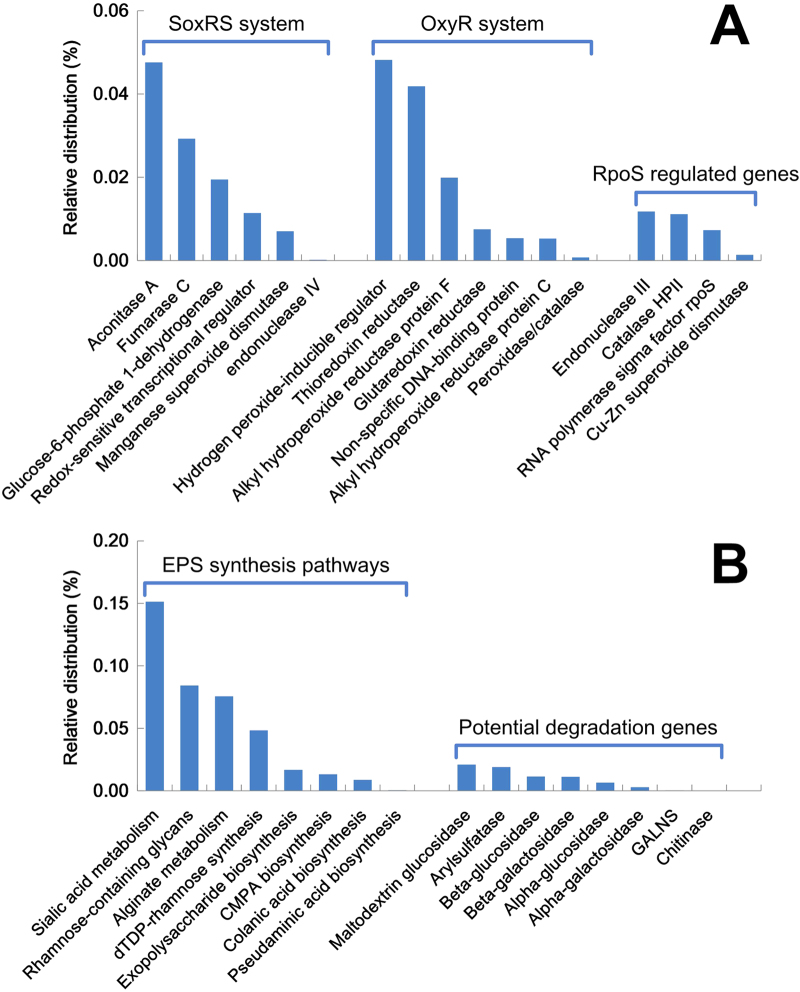
Relative abundances of genes related to the SoxRS system, the OxyR system and RpoS regulated genes (A), and EPS production and degradation (B), in the DW biofilm metagenome. Here, CMPA represents CMP-N-acetylneuraminate and GALNS represents N-acetylgalactosamine 6-sulfate sulfatase.

In addition to GSH metabolism, other oxidative stress response functions were analysed in the present study, including the SoxRS system, the OxyR system, and RpoS regulated genes (Fig. 6A). Several genes, such as aconitase A (*acnA*) and fumarase C (*fumC*) of the SoxRS system, and hydrogen peroxide-inducible regulator (*oxyR*) and thioredoxin reductase (*trxB*) of the OxyR system, were detected at greater abundances than other genes in both systems. In regard to the RpoS system, several genes were also detected at relatively low abundances in the metagenome of the DW biofilm (Fig. 6A).

The genes for EPS production and degradation in DW biofilms were also investigated (Fig. 6B) because EPS are also considered to play significant roles in the protection of DW biofilms against the residual disinfectants in DWDS^21^. Several EPS synthesis pathways, which primarily belong to cell wall and capsule subsystems, were identified in the DW biofilm. Among them, the genes for sialic acid synthesis were present in the greatest abundances, followed by rhamnose-containing glycans and alginate. Several enzymes that might be related to EPS degradation were detected in the metagenome of DW biofilm (Fig. 6B), including hydrolases, lyases, sulfatases, glycosidases and chitinases^22^.

### Genomic binning of the DW biofilm metagenome

After selection based on the coverage and tetranucleotide frequencies (TNFs), a total of 327 contigs were extracted to construct a draft genome of an ~3.65 Mb size. The completeness and redundancy of this draft genome were further evaluated. According to the core Cluster of Orthologous Groups (COGs) (Table S2), the completeness of the extracted draft genome was 99%, by comparison with the 96 reference genomes in the order of *Rhizobiales*, suggesting that the draft genome had a high similarity with the bacteria in this order. This similarity was further supported by the comparison of the core COGs between draft genome and the references from 9 families of the order *Rhizobiales*, with relatively high estimated completeness (78-91%, Table S2). Moreover, the essential single copy genes (ESCGs) in the draft genome were extracted and compared with that of *Alphaproteobacteria*. The results suggest that the completeness was greater than 95%; 100 out of 105 ESCGs were identified in the draft genome. For both methods, relatively low redundancy was observed (~3% redundancy for ESCGs, Table S2), suggesting that the draft genome had low contamination from other species.

**Figure 7:**
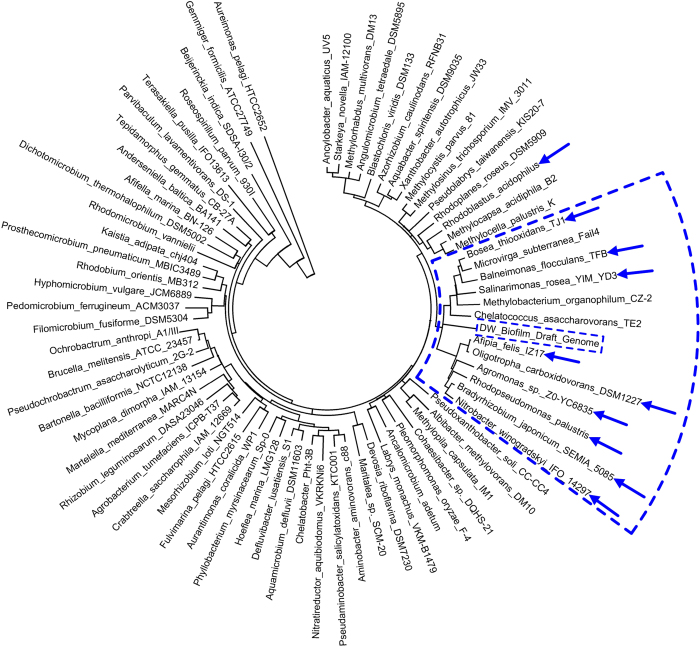
Phylogenetic tree of the draft genome and typical species in genera of the order *Rhizobiales*. The annular sector outlined by the blue dashed line indicates the cluster containing the draft genome. The blue arrows indicate the bacterial species that belong to the family *Bradyrhizobiaceae*. The 16S rRNA genes were compared by ClustalW multiple alignment. The phylogenetic tree was generated by the neighbour-joining method, with 1,000 of bootstrap replications, using the MEGA software (v 5.05).

A 16S rRNA gene with a length of 851 bp was identified in the draft genome by aligning the contigs against the SILVA SSU database using BLAST. This 16S rRNA read was not complete because it was located at one end of an assembled contig with a length of 10,886 bp (Fig. S3). According to the identification by the RDP Classifier and a NCBI BLAST search, the 16S rRNA read belonged to the order of *Rhizobiales*. However, it could not be further assigned to a lower taxonomic level (such as family or genus). According to the size of draft genome (~3.7 Mb), the evaluated completeness (~95%), the sequencing depth of the metagenome (~6.1 G) and the mean coverage of the binned contigs (~450), the relative abundance of this draft genome in the DW biofilm was approximately 28%, which is consistent with the taxonomic analysis (25 ± 2.2% of the unclassified 454 reads belonged to the order *Rhizobiales*). Furthermore, a phylogenetic tree analysis suggested that this draft genome might be more closely related to the family *Bradyrhizobiaceae* because the majority of the bacteria (9 out of 12) in the cluster that contained the draft genome belonged to the *Bradyrhizobiaceae* family (Fig. 7). This finding is consistent with the results of the taxonomic classification of the open reading frames (ORFs) in the draft genome, which revealed the draft genome could be assigned to the *Rhizobiales* order and more specifically to the *Bradyrhizobiaceae* family (Table S3). Therefore, the obtained draft genome was defined as a *Bradyrhizobiaceae*-like bacterium.

The pathway of GSH metabolism in this draft genome was evaluated and constructed by aligning the ORFs in the draft genome against the NCBI nr database via BLAST (Fig. 5B). Compared with the GSH metabolism of the DW biofilm (Fig. 5A), fewer enzymes were identified in the draft genome, indicating that the *Bradyrhizobiaceae*-like bacterium may have a specific pathway for GSH. Two enzymes for GSH synthesis were identified in the draft genome, including glutathione synthase and glutathione reductase. For GSH degradation, glutathione S-transferase (GST) and gamma-glutamyltranspeptidase were detected; in particular a greater number of GST genes (9 ORFs) were detected in the draft genome compared with other genes (1–3 ORFs) in the pathway (Fig. 5B). This finding might indicate that the detoxification process in this bacterium is mainly dependent on the GST pathway, which can catalyse the reaction of GSH with the substrates of toxic compounds (e.g., the residual chlorine in DW) and thus prevent these compounds from interacting with crucial cellular proteins or nucleic acids^23^.

## Discussion

Based on the taxonomic results, *Proteobacteria*, particularly *Alphaproteobacteria*, were dominant in the DW biofilms (Figs. 1 and 2). This finding is similar to the analytical results of several studies^6^,^8^, which also observed *Alphaproteobacteria* as dominant in DW biofilms. However, other studies have reported contrary results^11^,^15^, with *Betaproteobacteria*, rather than *Alphaproteobacteria*, as the most dominant class in their biofilms. This difference might be attributed to several explanations, including differences among applied molecular methods, variations in water qualities and pipe materials, and differences in the growth stages and the ages of the studied biofilms. In regards to the influences of pipe materials, slight differences in microbial community structure were observed between the biofilms grown on the two plastics (Fig. 2). Variation in the hydrophobicity interactions between the biofilm and the plastic substrates might have driven these insignificant differences because the physicochemical properties (Table S4) only vary significantly between PC and PE in terms of surface tension, and not surface charge or roughness. However, hydrophobicity interactions may not impose strong impacts on the development of DW biofilms. The results of present study indicate that SS, rather than plastics, may facilitate the formation of relatively higher diversity biofilms in DW systems (Table 1 and Fig. 1). This finding is supported by the results of other studies^24^,^25^,^26^ that have reported the development of a greater biofilm biomass on metals than on plastics. The difference in community structure between SS and plastics was substantially greater than that between the two plastics. This significant difference between types of material might be mainly attributed to the positive charge and high roughness of the SS surface (Table S4), which could increase the strength of the electrostatic force and the adhesion strength between bacterial cells and the SS surface^27^, thus facilitating the attachment of a greater diversity of bacteria on SS.

In the present study, the hypervariable V3-V4 region of the bacterial 16S rRNA gene was considered for taxonomic analysis because of its higher coverage over other regions of the 16S rRNA gene^28^. Compared with previous studies, the classified portions of the 454 reads in the 6 biofilms were acceptable (55%, on average at the genus level, Fig. S4) and were consistent with those of other studies examining similar hypervariable regions and read lengths^28^,^29^. However, there were still a number of unclassified species, especially for 180 d biofilms, indicating the unknown portion of microbial populations in DW biofilms. The relatively high proportions of unclassified reads at the family (53% and 50%) and genus (67% and 65%) levels in the 180 d biofilms (Fig. S4) are mainly attributed to the unclassified reads in the order *Rhizobiales* (the draft genome from the metagenomic binning), which accounted for 25% of the total 454 reads. Moreover, the detection of several new genera in the DW biofilms (Fig. 3) indicates that HTS techniques are promising tools for analysis of microbial communities in complex ecosystems at relatively high resolution.

GSH has been demonstrated to effectively enhance bacterial resistance to various chlorine compounds^30^ and has also been implicated in the regulation of other oxidation resistant systems, including the OxyR, SoxRS, and SOS systems^31^. Notably, the stresses from chlorine disinfection^32^ and starvation^31^ could strongly stimulate the synthesis of GSH. This protection mechanism may explain the poor performance of residual chlorine in controlling bacterial regrowth in DWDS^33^ and the formation of biofilms^34^, especially considering the stimulation of the GSH by oligotrophic conditions and residual chlorine in DWDS (Table 2). Moreover, the transfer of GSH metabolism genes from bacteria to eukaryotes via the mitochondrial progenitor in the evolutionary history has been proposed^35^. This proposed scenario suggests that the *Alphaproteobacteria*, which are considered the modern relatives of the mitochondrial progenitor^36^, commonly carry GSH related genes and, thus, have relatively high resistance to the disinfection processes of DW treatment plants and the residual chlorine in the DWDS. This hypothesis is consistent with the taxonomic observation of the present study, which demonstrated that *Alphaproteobacteria* dominated the DW biofilms (Fig. 2).

Several key genes involved in responses to oxidative stress were detected in relatively high abundance in the DW biofilm (Fig. 6A), including *acnA* and *fumC* in the SoxRS system, as well as *oxyR* and *trxB* in the OxyR system. The *acnA* and *fumC* genes are TCA cycle genes and could be directly activated by SoxRS^37^. Then, the TCA cycle is operating to produce reducing compounds to maintain cellular redox balance^38^. In addition to the TCA cycle, the SoxRS system can also activate glucose-6-phosphate 1-dehydrogenase (*zwf*) to produce NADPH/NADH in response to severe oxidative stress^38^. The results of the present study suggest that the SoxRS system in the DW biofilm mainly worked via the TCA cycle, rather than NADPH/NADH synthesis, according to the abundances of *acnA*, *fumC* and *zwf* in the DW biofilm metagenome (Fig. 6A). In addition to the SoxRS system, oxidative stress can also activate OxyR by oxidizing two cysteines and forming a reversible disulfide bond^39^. The activated OxyR can then induce the transcriptions of various antioxidant genes^38^, e.g., thioredoxin reductase (*trxB*), alkyl hydroperoxide reductase (*ahpCF*), glutaredoxin reductase (*grxA*), a non-specific DNA-binding protein (*dps*), and peroxidase/catalase (*katG*). Among these constituent genes of the OxyR system, *trxB* was the most abundant gene in the DW biofilm (Fig. 6A). This finding is consistent with the metagenome generated from a chlorinated DW sample^21^, revealing that *trxB* might play a key role in the OxyR system in DW ecosystems. Compared with the SoxRS and OxyR systems, in general, RpoS regulated genes were detected at relatively lower abundances. One explanation for this lower abundance might be that the RpoS genes most likely originated from the *Gammaproteobacteria*^40^, which were not dominant (16 ± 9.5%) in the DW biofilm, as previously mentioned (Fig. 2). In general, diverse oxidative stress responses were identified, indicating that the DW biofilm responded to residual disinfectants by activating multiple antioxidant systems rather than depending on a single mechanism for protection^41^.

Several EPS synthesis genes were detected in abundance in the DW biofilms, e.g., sialic acid and rhamnose-containing glycans (Fig. 6B). The sialylation of lipooligosaccharides containing sialic acid is indispensable for the biofilm formation of several pathogens^42^. Although the genes related to sialic acid synthesis are frequently found in abundance in biofilms^22^, the roles of sialic acid in biofilm formation for nonpathogenic bacteria remain unclear. Rhamnolipids are known to play various roles in *P. aeruginosa* biofilm formation and development^43^, including enhancing microcolony formation, facilitating bacterial migration and the development of mushroom-shaped structures, and preventing the colonization of channels. Several enzymes that may be related to EPS degradation were also detected in the metagenome of the DW biofilm (Fig. 6B). These EPS degrading enzymes have been proven to be important or even critical to biofilms. For instance, a number of enzymes can degrade biopolymers into low-molecular-mass products to provide additional carbon and energy sources for embedded bacteria^43^. Moreover, several enzymes are frequently involved in the degradation of structural EPS to facilitate the dispersion of bacterial cells from the biofilm matrix, likely in response to nutrient starvation^44^ or the requirement for the modification of the matrix by other bacteria^45^.

Genomic binning using metagenomic data have been widely applied to explore the complete and pure genomes of uncultured microorganisms in mixed communities in various ecosystems^46^,^47^,^48^,^49^. In the present study, according to the coverage of reads and TNFs of contigs, a near-complete and low-contamination draft genome of a *Bradyrhizobiaceae*-like bacterium was constructed from the metagenome of the DW biofilm. Similar results have been reported in other studies^6^,^11^,^15^, which also detected abundant bacteria in several genera of the *Bradyrhizobiaceae* family, such as *Afipia*, *Bosea* and *Bradyrhizobium*, in DW biofilms by analysing 16S rRNA genes. This finding suggests that genomic binning provides an effective supplementary tool in taxonomic analyses based on 16S rRNA genes^50^. Moreover, the acquisition of a draft genome provides a basis for the analysis of the functions of this bacterium and the further evaluation of its role in the development of the DW biofilm, especially considering its high abundance (~25%).

## Methods

### Biofilm samples

Four rotating annular reactors (BioSurface Technologies Corp., US) with 1.0 L of working volume were operated in parallel with a continuous flow of tap water in darkness to simulate a DWDS^51^. The rotating annular reactor is a simple apparatus designed to provide different coupons surfaces upon which a DW biofilm is cultivated under fluid hydrodynamic conditions that simulate the real environment of a DWDS. Rotating annular reactors have been successfully employed to study various aspects of DW biofilms, such as dynamics of biofilm growth^52^, efficiency of disinfection^34^, survival of pathogenic microorganisms^53^, and effects of pipe materials^8^. All of the reactors were disinfected using 1.2% sodium hypochlorite for 1 h before the experiments were established. The basic physicochemical properties of the tap water were tested bi-weekly (Table 2 and Table S5). The flow rate for each reactor was controlled at 16 mL/min, resulting in a hydraulic retention time of 1.0 h. The inner drums were rotated at a speed of 50 rpm, corresponding to a flow velocity of 0.3 m/s in a smooth pipe with diameter of 100 mm^8^. Three of the reactors were installed with polycarbonate (PC) coupons (11.5 × 37.0 mm^2^) and were used for a time course study of biofilm formation after 60, 120 and 180 d. The other reactor contained 36 coupons of polyethylene (PE) and 36 coupons of stainless steel (SS) that were cultivated for 180 d to evaluate the biofilm community development on different materials (Fig. S5), selected because they are widely used in DWDS. The coupons of the same material were removed from the drums, pooled together and immersed in 100 mL of ultrapure water containing 0.1% of Tween 80 and treated by 10 min of ultrasonication (B 8200E-1, Branson Ultrasonics Corporation, US) to detach the biofilm. The detached cells were collected from the water by filtration through a sterilized mixed cellulose esters membrane with a pore size of 0.22 μm (MVGSWG124, Millipore, US). The membranes were stored at –20 °C before DNA extraction. Hence, five DW biofilm samples were collected and designated as 60 d, 120 d, 180 d, PE and SS. To evaluate the experimental repeatability, the 180 d sample was split, filtered through two membranes, and analysed as technical duplicates (180 d_1 and 180 d_2).

### DNA extraction and PCR amplification

Genomic DNA was extracted from the 6 DW biofilm samples using a FastDNA^®^ SPIN Kit for Soil (MP Biomedicals, France) according to the manufacturer’s instruction. The concentration and purity of DNA were determined using a NanoDrop spectrophotometer (ND-1000, Thermo Fisher Scientific, US). The extracted DNA was stored at –20 °C until further treatments.

For PCR amplification, the hypervariable V3-V4 region of the bacterial 16S rRNA gene was amplified using a forward primer (5’-ACTCCTACGGGAGGCAGCAG-3’) and a cocktail of four equally mixed reverse primers, including R1 (5’-TACCRGGGTHTCTAATCC-3’), R2 (5’-TACCAGAGTATCTAATTC-3’), R3 (5’-CTACDSRGGTMTCTAATC-3’) and R4 (5’-TACNVGGGTATCTAATCC-3’)^54^. Barcodes were added at the 5’ terminus of the forward primer to allow for sample multiplexing during sequencing^55^. Three replicated 50 μL PCR reaction solutions were prepared, containing 25 μL of *Premix Ex Taq*^TM^ (TaKaRa, China), 2  μL of 10 μM forward and reverse primers, 50 ng of extracted DNA, and RT-PCR grade water (Ambion Inc., US). The thermocycling steps for PCR were set as follows: initial denaturation at 94 °C for 4 min, 30 cycles at 94 °C for 30 s, 50 °C for 45 s, 72 °C for 90 s, and a final extension step at 72 °C for 10 min. The PCR products of three replicates were combined and purified using a PCR quick-spin^TM^ PCR Product Purification Kit (iNtRON Biotechnology, Korea). The PCR products of the 6 samples were visualized on an agarose gel, quantified by Nanodrop, and stored at -20 °C.

### 454 pyrosequencing and Illumina sequencing

For 454 pyrosequencing, purified PCR products were sequenced using an FLX Titanium instrument (Roche). The acquired sequences were analysed using the Quantitative Insights into Microbial Ecology (QIIME v.1.3.0) pipeline^56^. The sequencing data were initially de-multiplexed and separated into their respective samples based on their nucleotide barcodes. Then, the sequences in each sample were denoised using AmpliconNoise (implemented in QIIME) with the default parameters, except that the Perseus algorithm for chimaera removal was disabled. Chimaera checking was performed using Chimera Slayer^57^. Finally, 66,388 cleaned 454 reads with a mean length of 438 ± 16 bp were obtained from the 6 DW biofilm samples.

The genomic DNA extracted from the 180 d sample was used for metagenome generation by Illumina sequencing. In detail, DNA fragmentation was performed using Covaris S2 (Covaris, 01801-1721). According to manufacturer’s instructions, the fragments were then processed by end reparation, A-tailing, adapter ligation, and DNA size-selection, before PCR reaction and product purification. Finally, a library of ~180 bp DNA fragment reads was constructed and then sequenced using an Illumina HiSeq 2000 instrument (BGI, China). The base-calling pipeline (Version Illumina Pipeline −0.3) was used to process the raw fluorescence images and call reads. Raw reads with >10% unknown nucleotides or with >50% low quality nucleotides (quality value <20) were discarded^58^. Finally, 6.1 Gb of cleaned data with read lengths of 100 bp were generated, containing 65,280,000 Illumina reads.

The Illumina platform occasionally produces a large number of reads that are nearly identical^59^; thus, de-replication was conducted using a self-written script. Only one representative read for each cluster of replicated reads (the first 50 bp of each cluster were identical) was used for the subsequent analysis. After de-replication, filtered reads were merged to form “tags” (with a minimum merging length of 10 bp) using another self-written python script to increase the annotation accuracy. Finally, 19,167,904 tags were generated with a mean length of 159 ± 9 bp and used for functional analysis.

### Taxonomic analysis

After removing the barcodes and primers using a self-written script, the sequence number of 454 reads was normalized by extracting the first 8,951 reads from each sample for all of the following analyses^55^. Then, the normalized samples were individually classified using the RDP Classifier (v 2.5)^60^. A confidence threshold of 80%, as suggested by the RDP, was applied to assign the reads to taxonomic levels.

### Diversity analysis

The diversity analysis of the DW biofilms was conducted using relevant tools in RDP. First, the pyrosequencing aligner was used to individually align the 454 reads of each sample using Infernal aligner^61^. Second, the aligned reads were clustered at cluster distances of 0.03 and 0.06 by applying the complete linkage clustering method. Finally, the Chao1 richness, Shannon diversity index, Pielou’s evenness index, and rarefaction curves for the DW biofilms were calculated using the corresponding analytical tools in RDP.

### Functional analysis

The microbial functions of the DW biofilm metagenome were analysed using the SEED subsystems^62^ and KEGG databases^20^ on the MG-RAST v3.2.5 sever^63^, employing an E-value of 1 × 10^−5^, a minimum identity of 60%, and a minimum alignment length of 15 aa. The results of the SEED subsystems were compared with 30 metagenomes from three aquatic ecosystems published on MG-RAST (Table S6) by PCA analysis using the PAST software (http://folk.uio.no/ohammer/past/, v 1.99). The GSH pathway of the DW biofilm was constructed by KEGG analysis of the *Bacteria* domain and was used to evaluate the specific reactions for GSH metabolism. The SoxRS system, the OxyR system, RpoS regulated genes, and EPS metabolism genes in DW biofilm were evaluated via SEED database.

### Genomic binning

After de-replication, *de novo* assembly was conducted to assemble the Illumina reads using the CLC software (v 6.0.2), with a k-mer size of 63^49^. The contigs longer than 300 bp were reserved for the bin construction. Then, contigs with a coverage between 300 and 600-fold coverage were selected for genomic binning because there was an obvious cluster of contigs at ~450-fold coverage (Fig. S6). The extracted contigs were further filtered by the MetaCluster algorithm based on the TNFs^64^. The completeness and redundancy of the acquired draft genome were evaluated by comparing the bin contigs with the core COGs of *Rhizobiales*^65^ and the ESCGs of *Alphaproteobacteria*^49^. MetaGeneMark (v 1.0) was applied to predict ORFs from the draft genome^66^. The predicted ORFs were compared against the NCBI nr database using an E-value of 1 × 10^−5^ and a minimum alignment length of 50 aa^67^. The results were imported into MEGAN (v 4.70.4) for functional analysis using the default parameters and the lowest common ancestor (LCA) algorithm^68^.

## Additional Information

**How to cite this article**: Chao, Y. *et al.* Diversity and functions of bacterial community in drinking water biofilms revealed by high-throughput sequencing. *Sci. Rep.* **5**, 10044; doi: 10.1038/srep10044 (2015).

## Supplementary Material

Supporting Information

## Figures and Tables

**Table 1:** Diversity analysis of DW biofilm samples using the RDP pipeline.

Sample	0.03 distance			0.06 distance		
	Chao1	Shannon	Pielou	Chao1	Shannon	Pielou
60 d	382	2.9	0.55	303	2.8	0.55
120 d	386	3.1	0.64	339	3.0	0.66
180 d_1	350	2.9	0.52	280	2.8	0.53
180 d_2	393	3.0	0.55	328	2.9	0.55
PE	648	3.8	0.63	524	3.6	0.62
SS	1,794	4.4	0.65	1,372	4.2	0.64

**Table 2:** Water quality of the tap water used in the present study (n = 12).

Parameter (Units)	Mean	SD a	Max	Min
Temperature (°C)	25	1.5	27	22
pH	7.3	0.35	7.7	6.6
Dissolved oxygen (mg/L)	7.1	0.33	7.7	6.7
Total organic carbon (mg/L)	1.9	0.51	2.7	1.1
Inorganic carbon (mg/L)	6.4	0.79	7.7	5.4
Total nitrogen (mg/L)	1.4	0.41	2.1	0.98
Total phosphate (mg/L)	0.010	0.0055	0.021	0
Residual chlorine (mg/L)	0.066	0.044	0.13	0
Conductivity (μs/cm)	154	13	173	136
Turbidity (NTU b)	0.25	0.23	0.88	0.084
Total dissolved solid (mg/L)	140	38	212	88

^a^Standard deviation;

^b^Nephelometric turbidity units.
