# Supplementary material for: Diversity and functions of bacterial community in drinking water biofilms revealed by high-throughput sequencing
Source: Sci Rep. 2015 Jun 12;5:10044. doi: 10.1038/srep10044 (PMC4464384; doi:10.1038/srep10044)
Supplement: Supporting Information [file srep10044-s1.pdf]

Supporting Information

For

Diversity and functions of bacterial community in drinking water biofilms revealed by high-throughput sequencing

Yuanqing Chao[1,2], Yanping Mao[1], Zhiping Wang[1,3], Tong Zhang[1,*]

[1] Environmental Biotechnology Lab, The University of Hong Kong, Pokfulam Road, Hong Kong SAR, China

[2] School of Environmental Science and Engineering, Sun Yat-Sen University, Guangzhou, China

[3] School of Environmental Science and Engineering, Shanghai Jiao Tong University, Shanghai, China

[*] Corresponding author. Email: zhangt@hku.hk. Phone: (852) 28598551. Fax: (852) 25595337.

Experimental repeatability

To test the repeatability of DNA extraction, PCR and 454 pyrosequencing, the 180 d biofilm was divided into duplicates. DNA was extracted from the two duplicates, PCR conducted and then sequenced individually. The results generated indicated that the analysis was repeatable at the genus level (R^2 = 0.976, Figure S7).

Table S1 The genus list of several bacterial groups, including potentially pathogenic bacteria, iron-oxidizing bacteria, and sulfate-reducing bacteria, according to previous studies.

Bacterial groups	Potentially pathogenic bacteria	Iron-oxidizing bacteria	Sulfate-reducing bacteria
Genus list	*Aeromonas*	*Acidimicrobium*	*Bdellovibrio*
	Arcobacter	*Acidithiobacillus*	*Clostridium*
	Bordetella	*Alicyclobacillus*	*Desulfobacter*
	Borrelia	*Aquifex*	*Desulfobacterium*
	Brucella	*Chlorobium*	*Desulfobulbus*
	Campylobacter	*Ferrimicrobium*	*Desulfococcus*
	Chlamydia	*Ferrithrix*	*Desulfohalobium*
	Chlamydophila	*Ferritrophicum*	*Desulfomicrobium*
	Clostridium	*Gallionella*	*Desulfomonas*
	Corynebacterium	*Leptospirillum*	*Desulfomonile*
	Enterobacter	*Leptothrix*	*Desulfonatronum*
	Enterococcus	*Marinobacter*	*Desulfonema*
	Escherichia	*Mariprofundus*	*Desulfosarcina*
	Francisella	*Pseudogulbenkiania*	*Desulfotomaculum*
	Haemophilus	*Rhodobacter*	*Desulfovibrio*
	Helicobacter	*Rhodopseudomonas*	*Desulfuromonas*
	Klebsiella	*Sideroxydans*	*Desulfuromusa*
	Legionella	*Sphaerotilus*	*Geobacter*
	Leptospira	*Thiobacillus*	*Myxococcus*
	Listeria		*Pelobacter*

	Mycobacterium		*Pseudomonas*
	Mycoplasma		*Syntrophobacter*
	Neisseria		*Thermodesulfobacterium*
	Pseudomonas		*Thermodesulfovibrio*
	Salmonella		
	Serratia		
	Shigella		
	Staphylococcus		
	Streptococcus		
	Treponema		
	Vibrio		
	Yersinia		
Total	32	19	24
Reference	Ye and Zhang, 2011	Emerson *et al*., 2010	Castro *et al*., 2000

Reference:

Ye, L. & Zhang, T. Pathogenic bacteria in sewage treatment plants as revealed by 454 pyrosequencing. *Environ. Sci. Technol.* **45,** 7173-7179 (2011).

Emerson, D., Fleming, E. J. & McBeth, J. M. Iron-oxidizing bacteria: an environmental and genomic perspective. *Annu. Rev. Microbiol.* **64,** 561-583 (2010).

Castro, H. E., Williams, N. H. & Ogram, A. Phylogeny of sulfate-reducing bacteria. *FEMS Microbiol. Ecol.* **31,** 1-9 (2000).

Table S2 Evaluation of completeness and redundancy of extracted draft genome by core COGs of the reference genomes in order *Rhizobiales* and 9 families of this order.

Levels	Name	No. of Reference	Core COGs		Single core COGs		Completeness (%)	Redundancy (%)
			Bin	Reference	Bin	Reference		
Order	*Rhizobiales*	96	74	75	0	0	99	0
	Bartonellaceae	9	655	725	514	573	90	19
	Beijerinckiaceae	2	1,169	1,487	702	914	79	13
	Bradyrhizobiaceae	16	1,033	1,195	0	1	86	0
	Brucellaceae	21	1,055	1,350	607	808	78	10
Family	*Hyphomicrobiaceae*	5	928	1,045	481	548	89	7
	Methylobacteriaceae	8	1,190	1,539	479	637	77	5
	Phyllobacteriaceae	6	1,095	1,287	476	561	85	5
	Rhizobiaceae	24	573	629	243	275	91	3
	Xanthobacteraceae	3	1,174	1,460	588	736	80	8

Table S3 Phylogenetic analysis of the draft genome by annotating the ORFs.

Level	Closest taxon [a]	Attributed ORFs	Percentage	Criteria [b]	Pass/Fail
Phylum	*Proteobacteria*	3,178/3,458	92%	>50%	Pass
Class	*Alphaproteobacteria*	2,641/3,458	76%	>50%	Pass
Order	*Rhizobiales*	1,568/3,458	45%	>40%	Pass
Family	*Bradyrhizobiaceae*	618/3,458	18%	>34%	Fail

[a] The closest taxon at a specific level was selected according to the number of attributed ORFs;

[b] The phylogenetic levels of bins were identified by the following criteria: for phylum and class level, > 50% of ORFs in the draft genome could be attributed; for order and family level, > 40% and > 34% of ORFs could be attributed, respectively (Ishii et al., 2013) .

Reference:

Ishii, S. et al. A novel metatranscriptomic approach to identify gene expression dynamics during extracellular electron transfer. Nat. Commun. **4,** 1601 (2013).

Table S4 Surface tension, zeta potential and RMS roughness of the three materials used in the present study.

Materials	Surface tension (mJ/m^2) [a]	Zeta potential (mV) [b]	RMS roughness (nm) [c]
PC	42 ± 1.1	-27 ± 4.6	21 ± 8.5
PE	34 ± 1.6	-29 ± 2.3	21 ± 6.4
SS	41 ± 1.8	40 ± 7.3	83 ± 25

[a] Surface tension was evaluated by using contact angle method;

[b] Zeta potential was measured in 100 mM PBS solution;

[c] Roughness was calculated by the AFM height images with scan sizes of 2 × 2 μm^2.

Table S5 Detailed water quality of the tap water in all 12 time points.

Parameters (Unit)	Time points												Average ± SD [a]
	1	2	3	4	5	6	7	8	9	10	11	12	
Temperature (℃)	27	26	26	22	23	25	23	26	25	25	26	26	25 ± 1.5
pH	7.1	7.5	6.9	7.4	7.7	6.6	7.1	7.4	7.7	7.5	7.0	7.7	7.3 ± 0.35
Dissolved oxygen (mg/L)	6.9	7.5	7.0	6.8	7.7	7.2	7.1	6.8	7.0	7.6	6.7	7.4	7.1 ± 0.33
Total organic carbon (mg/L) [b]	1.1	2.6	1.9	2.7	1.7	1.3	1.5	2.3	1.3	2.0	2.1	2.3	1.9 ± 0.51
	1.1	2.5	1.7	2.5	1.8	1.2	1.5	2.3	1.4	1.9	2.1	2.5	
Inorganic carbon (mg/L) [b]	7.0	6.1	5.5	6.2	5.6	7.6	5.8	6.1	6.8	7.2	7.6	5.5	6.4 ± 0.79
	7.0	6.1	5.4	6.2	5.7	7.7	5.8	5.9	7.0	7.2	7.5	5.5	
Total nitrogen (mg/L)	2.1	1.4	1.1	2.1	1.0	1.3	0.98	1.2	1.2	1.6	1.9	1.1	1.4 ± 0.41
Total phosphate (mg/L)	0.021	0.0084	0.0084	0.0084	0.017	0.013	0.013	0.013	0.0084	0	0.0084	0.0042	0.010 ± 0.0055
Residual chlorine (mg/L)	0.02	0.08	0.1	0.11	0.07	0.09	0.11	0	0.04	0.13	0.02	0.02	0.066 ± 0.044
Conductivity (µs/cm)	154	144	139	136	148	152	166	170	160	173	170	139	154 ± 13
Turbidity (NTU [c])	0.88	0.48	0.26	0.19	0.12	0.12	0.20	0.29	0.17	0.11	0.084	0.097	0.25 ± 0.23
Total dissolved solid (mg/L)	150	146	88	136	152	108	128	212	100	184	179	102	140 ± 38

[a] Standard deviation; [b] TOC and IC tests were repeated twice for one sample; [c] Nephelometric turbidity units.

Table S6 The basic information of 30 metagenomic data in three aquatic ecosystems (including ocean, fresh water and drinking water), which were applied to principal component analysis in the present study.

Biome	MG-RAST ID	Metagenome size (bp)	Reads No.	Location	Sequence Method
Ocean_1	4441148.3	54,752,102	50,609	Indian Ocean, St. Anne Island, Seychelles	other [a]
Ocean_2	4441139.4	52,667,848	50,096	Indian Ocean, International waters between Madagascar and South Africa	other
Ocean_3	4441150.3	64,230,062	61,020	Indian Ocean	other
Ocean_4	4441155.3	62,752,349	59,813	Indian Ocean	other
Ocean_5	4441608.3	53,607,277	49,597	Indian Ocean	other
Ocean_6	4441135.3	45,710,196	46,052	Indian Ocean, Madagascar Waters, Madagascar	other
Ocean_7	4441149.3	64,223,447	60,932	Outside Seychelles, Indian Ocean, Seychelles	other
Ocean_8	4441147.3	55,638,894	52,118	Indian Ocean	other
Ocean_9	4441156.3	62,072,289	59,080	Indian Ocean	other
Ocean_10	4441134.3	53,607,277	49,597	Indian Ocean	other
Fresh water_1	4534328.3	1,888,723,700	18,887,237	Minnesota, United States	Illumina
Fresh water_2	4534330.3	1,847,964,400	18,479,644	Minnesota, United States	Illumina
Fresh water_3	4534342.3	1,647,353,500	16,473,535	Minnesota, United States	Illumina
Fresh water_4	4534350.3	1,623,038,100	16,230,381	Minnesota, United States	Illumina

Fresh water_5	4534344.3	1,606,029,200	16,060,292	Minnesota, United States	Illumina
Fresh water_6	4534338.3	1,599,106,200	15,991,062	Minnesota, United States	Illumina
Fresh water_7	4534336.3	1,430,592,200	14,305,922	Minnesota, United States	Illumina
Fresh water_8	4534340.3	1,427,643,200	14,276,432	Minnesota, United States	Illumina
Fresh water_9	4534346.3	1,395,406,900	13,954,069	Minnesota, United States	Illumina
Fresh water_10	4534348.3	1,031,183,000	10,311,830	Minnesota, United States	Illumina
DW disinfected_1	4470937.3	86,319,144	155,593	Cincinnati, Ohio, United States	454
DW disinfected_2	4470954.3	197,354,490	362,640	Cincinnati, Ohio, United States	454
DW disinfected_3	4509542.3	1,191,805,800	11,918,058	Hong Kong SAR, China	Illumina
DW disinfected_4	4509336.3	3,455,504,600	34,555,046	Hong Kong SAR, China	Illumina
DW disinfected_5	4615185.3	1,193,976,400	11,939,764	Nanjing, China	Illumina
DW undisinfected_1	4509541.3	1,190,701,600	11,907,016	Hong Kong SAR, China	Illumina
DW undisinfected_2	4509333.3	4,192,434,200	41,924,342	Hong Kong SAR, China	Illumina
DW undisinfected_3	4509334.3	3,607,833,600	36,078,336	Hong Kong SAR, China	Illumina
DW undisinfected_4	4615186.3	1,191,712,400	11,917,124	Nanjing, China	Illumina
DW undisinfected_5	4615323.3	1,078,393,739	10,783,937	Nanjing, China	Illumina

[a] indicates the researchers applied other sequencing method, except for sanger, Illumina and 454 methods.

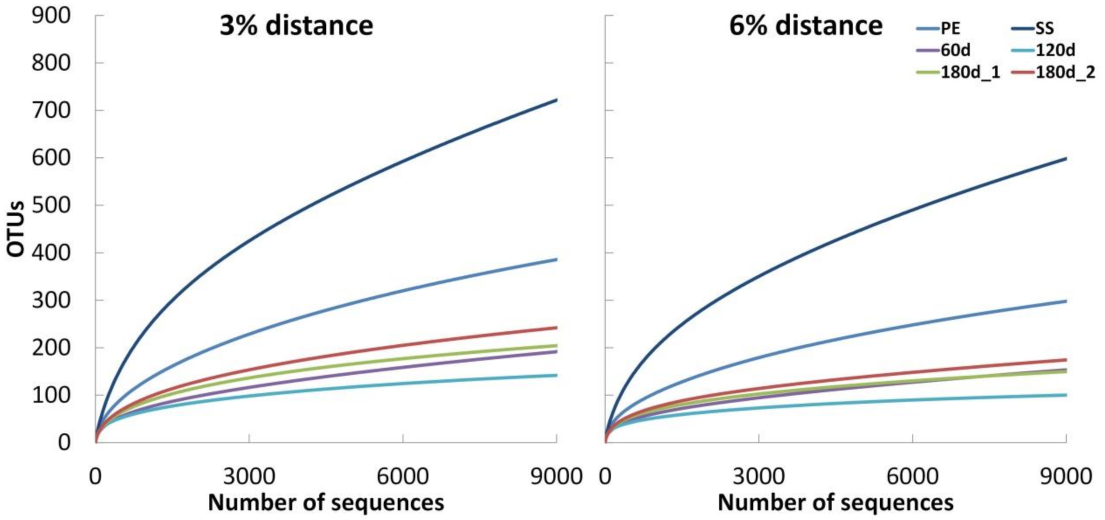

Figure S1 Rarefaction curves of OTUs defined by 0.03 and 0.06 cluster distances in the DW biofilm samples.

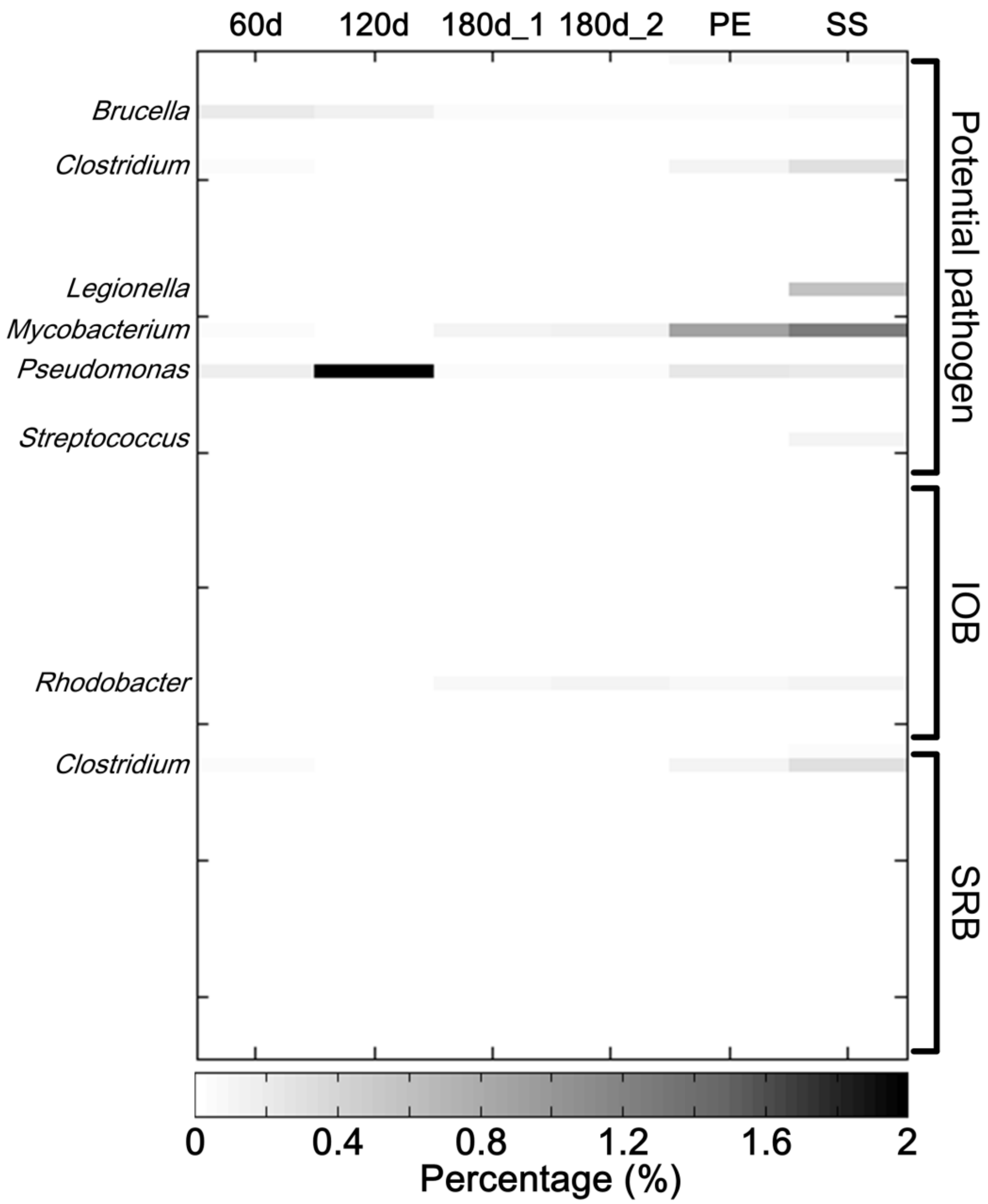

Figure S2 Relative distribution of 454 reads in several bacterial groups at the genus level, including potentially pathogenic bacteria, iron-oxidizing bacteria, and sulfate-reducing bacteria (Table S1).

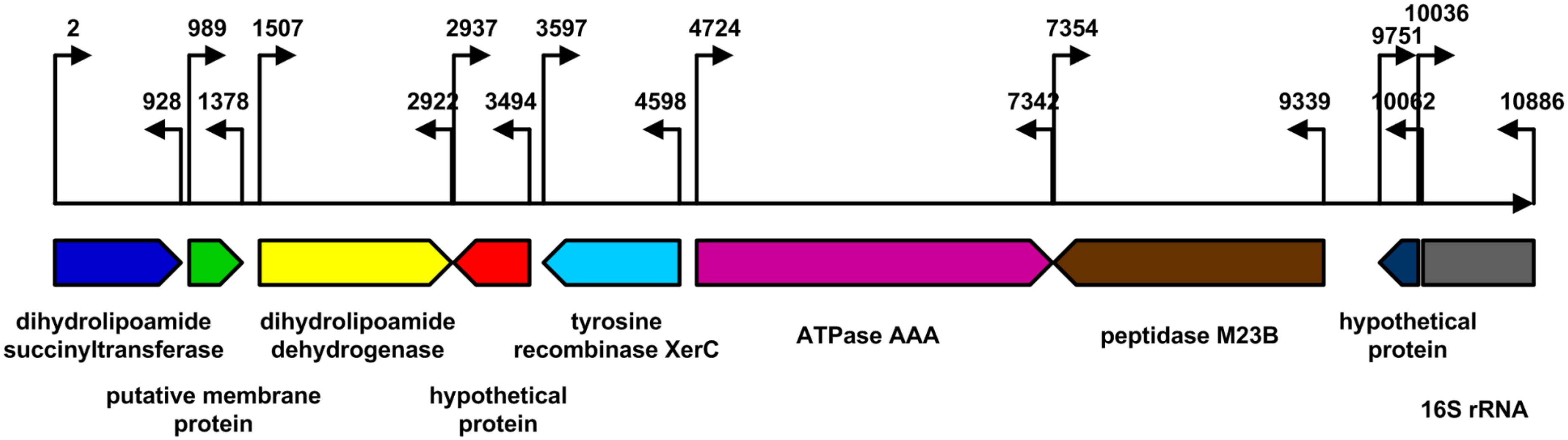

Figure S3 The contig which carried 16S rRNA in the draft genome.

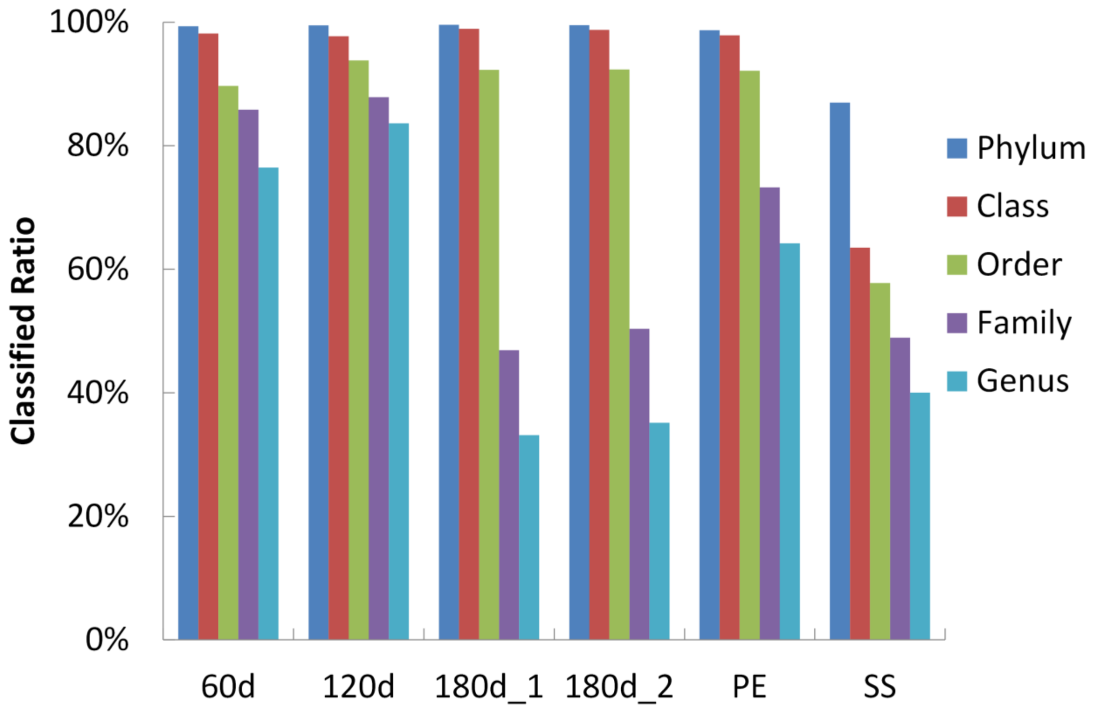

Figure S4 Classified ratio of 454 reads at different taxonomic levels, including phylum, class, order, family and genus, in the 6 biofilms by RDP.

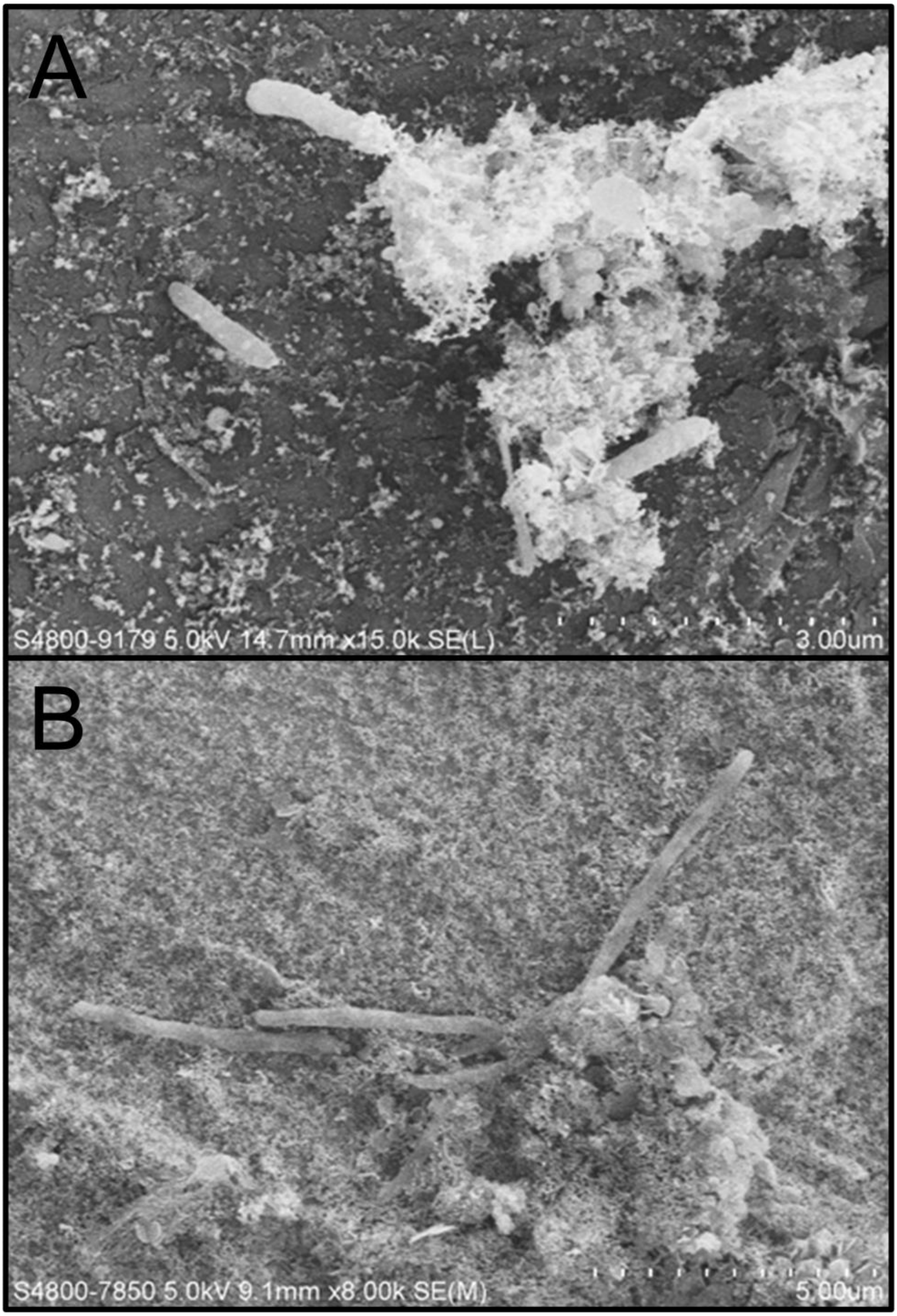

Figure S5 SEM images of the DW biofilms formed on PE (A) and SS (B).

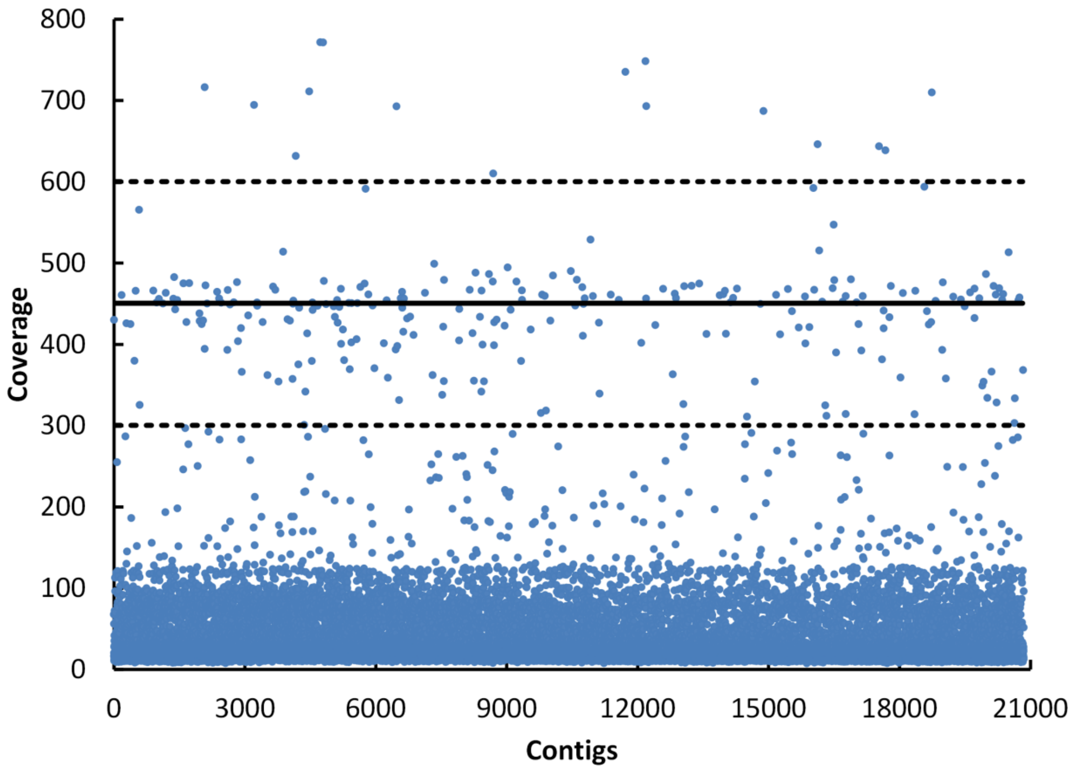

Figure S6 Coverage distribution of the assembled contigs in the DW biofilm metagenome.

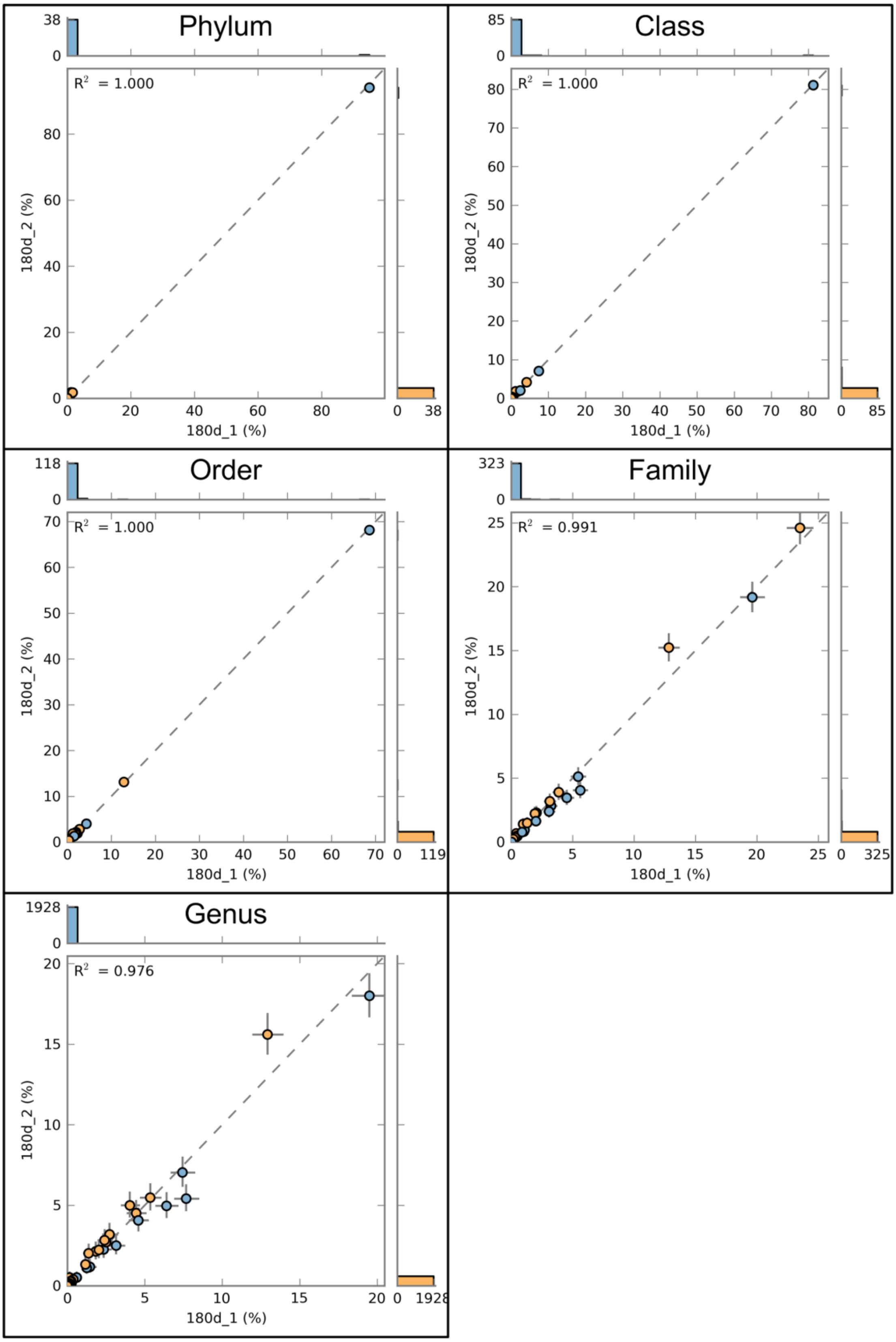

Figure S7 Repeatability evaluation of two duplicates, i.e. 180d_1 (blue) and 180d_2 (brown) biofilms, by Scatter plots using STAMP software.
